# Role of the TGF‐β pathway in dedifferentiation of human mature adipocytes

**DOI:** 10.1002/2211-5463.12250

**Published:** 2017-07-10

**Authors:** Julie Anne Côté, Julie Lessard, Mélissa Pelletier, Simon Marceau, Odette Lescelleur, Julie Fradette, André Tchernof

**Affiliations:** ^1^ Institut Universitaire de Cardiologie et de Pneumologie de Québec Canada; ^2^ Endocrinologie et Néphrologie CHU de Québec Canada; ^3^ École de Nutrition Université Laval Québec Canada; ^4^ Faculté de Médecine, Département de Chirurgie, Centre de recherche en organogénèse expérimentale de l'Université Laval/LOEX Université Laval Québec Canada; ^5^ Division de Médecine Régénérative CHU de Québec Canada

**Keywords:** adipocyte, ceiling culture methods, collagens, dedifferentiation, transforming growth factor beta

## Abstract

Dedifferentiation of adipocytes contributes to the generation of a proliferative cell population that could be useful in cellular therapy or tissue engineering. Adipocytes can dedifferentiate into precursor cells to acquire a fibroblast‐like phenotype using ceiling culture, in which the buoyancy of fat cells is exploited to allow them to adhere to the inner surface of a container. Ceiling culture is usually performed in flasks, which limits the ability to test various culture conditions. Using a new six‐well plate ceiling culture approach, we examined the relevance of TGF‐β signaling during dedifferentiation. Adipose tissue samples from patients undergoing bariatric surgery were digested with collagenase, and cell suspensions were used for ceiling cultures. Using the six‐well plate approach, cells were treated with SB431542 (an inhibitor of TGF‐β receptor ALK5) or human TGF‐β1 during dedifferentiation. Gene expression was measured in these cultures and in whole adipose tissue, the stromal–vascular fraction (SVF), mature adipocytes, and dedifferentiated fat (DFAT) cells. TGF‐β1 and collagen type I alpha 1 (COL1A1) gene expression was significantly higher in DFAT cells compared to whole adipose tissue samples and SVF cells. TGF‐β1, COL1A1, and COL6A3 gene expression was significantly higher at day 12 of dedifferentiation compared to day 0. In the six‐well plate model, treatment with TGF‐β1 or SB431542, respectively, stimulated and inhibited the TGF‐β pathway as shown by increased TGF‐β1, TGF‐β2, COL1A1, and COL6A3 gene expression and decreased expression of TGF‐β1, COL1A1, COL1A2, and COL6A3, respectively. Treatment of DFAT cells with TGF‐β1 increased the phosphorylation level of SMAD 2 and SMAD 3. Thus, a new six‐well plate model for ceiling culture allowed us to demonstrate a role for TGF‐β in modulating collagen gene expression during dedifferentiation of mature adipocytes.

**Abbreviations**
COL1A1: collagen type I alpha 1
COL1A2: collagen type I alpha 2
COL6A3: collagen type 6 alpha 3
DFAT: dedifferentiated
ECM: extracellular matrix
KRH: Krebs–Ringer‐Henseleit
OM: omental
SC: subcutaneous
SVF: stromal–vascular fraction
TGF‐β: transforming growth factor beta

Adipose tissue contains connective tissue matrix, preadipocytes, immune cells, and mature adipocytes 1. Mature adipocytes are specialized in lipid storage and are generated from the differentiation of mesenchymal stem cells committed to preadipocytes 2. Matsumoto *et al*. 3 demonstrated that adipocytes can dedifferentiate into precursor cells to acquire a fibroblast‐like phenotype using ceiling culture. This method is based on the buoyancy of adipocytes, which allows them to adhere to the top inner surface of a reversed culture flask that is completely filled with medium 4. In 2000, Zhang *et al*. 5 proposed another culture technique in which adipocytes adhere to the underside of a floating piece of glass. More recently, Jumabay *et al*. obtained dedifferentiated (DFAT) cells from adipocytes using a method that did not require attachment of the cells to a plastic surface as opposed to ceiling culture. In this experimental model, the isolated adipocytes are incubated in the culture medium for 24 h and then transferred to another dish containing a filter. After 5 days, the filter is removed and the DFAT cells sink through the filter to the bottom of the dish 6. We have recently described a modified version of the ceiling culture approach in six‐well plates, allowing us to decrease the number of cells used and test a larger number of culture conditions 7.

TGF‐β1 has often been described as an important regulator of adipocyte physiology because of its role in inhibiting adipogenesis 8, 9, 10. This growth factor also plays a major role in adipose tissue remodeling through the induction of ECM protein‐coding genes such as collagens 11, 12, 13. We have recently demonstrated that genes coding for proteins of the extracellular matrix including COL1A1, COL1A2, COL6A3 were significantly upregulated during the dedifferentiation process. In that analysis, we did not observe significant changes in gene expression of other types of collagens including COL4A3, COL5A1, COL5A2, COL6A1, COL6A2, COL8A2, COL20A1 14. Moreover, we have previously shown that gene expression of matrix metalloproteinase 1 (MMP1), fibroblast‐activated protein (FAP), dipeptidyl peptidase IV (DPP4), and transforming growth factor β1 (TGF‐β1) was strongly induced during dedifferentiation. Finally, recent data support a role for the TGF‐β pathway in the dedifferentiation of human pancreatic islet β cells *in vivo* 15.

Although many groups have used the ceiling culture approach, many aspects of this unique cellular process remain to be characterized. Our previous reports 7, 16 suggest that the TGF‐β pathway may be involved in the dedifferentiation process and contribute to the generation of a proliferative cell population that could be useful in cellular therapy or tissue engineering. From the technical standpoint, previous culture systems limited our ability to test various incubation conditions during the process. To the best of our knowledge, no study has ever attempted to modulate the molecular, metabolic, or secretory attributes of the cells during dedifferentiation. Our objective was to implement a new six‐well plate culture system and modulate the dedifferentiation process by targeting the TGF‐β pathway and its effects on the expression of collagens. We hypothesized that the TGF‐β pathway is a significant modulator of COL1A1, COL1A2, and COL6A3 gene expression during the dedifferentiation of mature adipocytes.

## Materials and methods

### Tissue sampling

We have complied with all mandatory laboratory health and safety procedures in the course of the experimental work presented in this paper. The project was approved by the Research Ethics Committee of the Institut Universitaire de Cardiologie et de Pneumologie de Québec (IUCPQ). Written informed consent was obtained from tissue donors prior to sampling through the management framework of the IUCPQ Obesity Tissue Bank. Adipose tissue samples were obtained from men and women undergoing bariatric surgery. Portions of adipose tissues were digested with collagenase type I in Krebs–Ringer‐Henseleit (KRH) buffer for up to 45 min at 37 °C according to a modified version of the Rodbell method 17. Adipocyte suspensions were filtered through nylon mesh and washed three times with KRH buffer to obtain a purified population of mature adipocytes. The residual KRH buffer of adipocyte isolation, which contained the stromal–vascular fraction, was centrifuged and the pellet was washed in DMEM‐F12 culture medium supplemented with 10% fetal bovine serum, 2.5 μg·mL^−1^ amphotericin B, and 50 μg·mL^−1^ gentamicin. The stromal–vascular cell fraction was then filtered through 140‐μm nylon mesh to remove endothelial/mesothelial cells, placed in culture plates, and cultured at 37 °C under a 5% CO_2_ atmosphere. Medium was changed every two to three days. Isolated mature adipocytes were used for ceiling culture, whereas the stromal–vascular fraction was seeded in standard culture flasks containing DMEM‐F12 culture medium supplemented with 2.5 % fetal bovine serum. In previous studies, we have demonstrated that adipocytes can successfully dedifferentiate independent of their fat depot origin (SC or OM). We also demonstrated that the dedifferentiation process is relatively independent of obesity level, sex, or age of the cell donor 14, 16. Consistent with these results, data from SC and OM samples were combined in our analyses.

### Ceiling culture

**Figure 1:**
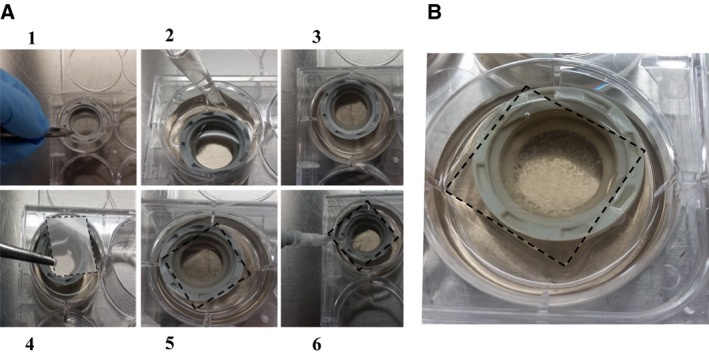
(A) Picture of the six‐well plate ceiling culture model. Panels 1, 2, and 3: 8 mL of DMEM/F12 20% fetal bovine serum was added to each well containing a 1/2‐inch plastic bushing; panels 4 and 5: A glass slide was put on top of the bushing; panel 6: Mature adipocytes were seeded under each coverslip. (B) Cells floated and adhered to the slides. They can be studied at specific time points.

When no treatment was used during the dedifferentiation process, isolated mature adipocytes were counted and 500 000 cells were added to a T‐25 flask completely filled with DMEM‐F12 culture medium supplemented with 20% fetal bovine serum, 2.5 μg·mL^−1^ amphotericin B, and 50 μg·mL^−1^ gentamicin. Flasks were incubated upside down at 37 °C, in 5% CO_2_ for 7 days. Cells floated up and adhered to the top inner ceiling surface of the flask. After seven days, the medium was removed and the flasks were inverted so that the cells were on the bottom until day 12 in the same medium 7. The medium was changed every three days. For gene expression analysis, cultures from each patient were harvested at days 4 and 7 of the dedifferentiation process as conducted previously 14. One flask per depot per patient was reversed at day 7 and maintained in culture for an additional 5 days (corresponding to day 12). Time points were chosen based on our observations that harvesting cells at day 4 provides a population of round cells that has completely adhered to the flask, while many cells at day 7 begin to be elongated. All ceiling cultures performed in the laboratory were reversed at day 7. Day 12 represents a time point at which the majority of cells have a fibroblast‐like morphology. When DFAT cells were needed for western blot experiments, cultures were maintained in standard condition for more than 12 days and subcultured when cells reached confluence. For other experiments, a ceiling culture model in six‐well plates was used 7. To do so, 8 mL of DMEM/F12 20% fetal bovine serum was added to each well containing a 1/2‐inch plastic bushing. A glass slide was put on top of the bushing and mature adipocytes were seeded under each coverslip (Fig. 1). These mature adipocytes floated and then adhered to the top slide. They were examined at specific time points during the dedifferentiation process. This model required a smaller number of cells and allowed us to use various media during the dedifferentiation process 7. The effectors were added to the media, and when appropriate, the glass slides with the adherent cells were flipped in a new well and then harvested for RNA extraction.

### RNA extraction and real‐time quantitative RT‐PCR

Total RNA was isolated from SVF cells, whole adipose tissue, and DFAT cells from five donors using the RNeasy lipid tissue extraction kit (Cat No./ID: 74804) and digested with RNase‐free DNase (Qiagen, Mississauga, ON, Canada) to remove all traces of DNA. RNA extraction was also performed from dedifferentiation time course experiments at day 0 (freshly isolated adipocytes), day 4, and day 7 of ceiling culture and at day 12 from three donors using the QIAGEN RNeasy extraction kit (Cat No. 330503) and in treated DFAT cells. To assess RNA quantity and quality, an Agilent Technologies 2100 Bioanalyzer and RNA 6000 Nano LabChip kit (Agilent, Mountain View, CA, USA) were used. For each sample, cDNA was obtained using the QuantiTect reverse transcriptase kit (Cat No 205311). The following sequences were used for quantitative PCR (forward/reverse): ATP synthase, H+ transporting, mitochondrial F1 complex, O subunit (ATP5O): 5′‐AACGACTCCTTGGGTATTGCTTAA‐3′/5′‐ATTGAAGGTCGCTATGCCACAG‐3′, glucose‐6‐phosphate dehydrogenase (G6PD): 5′ GCAGGGCATTGAGGTTGGGAG‐3′/5′‐GATGTCCCCTGTCCCACCAACTCTG‐3′, transforming growth factor β1 (TGF‐β1): 5′‐AAG TTG GCA TGG TAGCCC TT‐3′/5′‐CCC TGG ACA CCA ACT ATT GC‐3′, transforming growth factor β2 (TGF‐β2): 5′‐CTC CAT TGC TGA GAC GTC AA‐3′/5′‐ATA GAC ATG CCG CCC TTC TT‐3′, transforming growth factor β3 (TGF‐β3): 5′‐CAC ATT GAA GCG GAA AAC CT‐3′/5′‐AAA TTC GAC ATG ATC CAG GG‐3′, collagen type I alpha 1 (COL1A1): 5′‐CAC ACG TCT CGG TCA TGG TA3′/5′‐AAG AGG AAG GCC AAG TCG AG‐3′, collagen type I alpha 2 (COL1A2): 5′‐AGC AGG TCC TTG GAA ACC TT3′/5′‐GAA AAG GAG TTG GAC TTG GC‐3′, collagen type 6 alpha 3 (COL6A3): AAG TGC CGA TGT TTC CTC AT3′/5′‐TAA TTG AAT CGA GGA GCC CA‐3′. Housekeeping gene expression (ATP5O and G6PD) was measured in each sample. Results are expressed as ΔCt relative to housekeeping gene expression. Graph bars represent mean values of ΔCt values, and error bars are the standard error means (SEM). Only G6PD‐normalized results are shown but both housekeeping genes yielded similar results.

### TGF‐β1 recombinant treatment and TGF‐β receptor 1 inhibitor

Mature adipocytes were counted so that 50 000 cells were seeded in six‐well plates for ceiling culture in 20% serum. At day 4, slides with adherent adipocytes were reversed into a new plate containing 2 mL of 5% serum in each well. At day 5, cells (*n* = 7) were treated with 5 ng·mL^−1^ recombinant human TGF‐β1 or vehicle (0.1% bovine serum albumin) for 24 h (Ref Cat 100‐21) or with 1 μM SB431542 (*n* = 8) (Cat. No 1614), an inhibitor of the TGF‐β receptor ALK5 or with vehicle (dimethylsulfoxide, DMSO 20 mg·mL^−1^) for 24 h. The cells were then harvested in phenol buffer (Cat No./ID: 79306) for RNA extraction. Three replicates were cultivated for each condition and pooled together at day 6 into phenol buffer for RNA extraction and RT‐PCR quantification.

### Western blotting and antibodies

Proteins were extracted from the organic phase of the RNA phenol/chloroform extraction. First, 100% ethanol was added to the organic phase and incubated for 5 min. After centrifugation (4500 rpm, 2 min, 4 °C), the supernatant was incubated for 10 min with 1.5 mL isopropanol. The pellet was washed three times with 1.5 mL ethanol/0.3 m guanidine with 20‐min incubations and one additional wash without guanidine. The pellet was then incubated at 65 °C in Tris pH 7.4–6%/SDS until complete dissolution. Sonication was performed as a final disruption step. Protein samples (30 μg) were run on a 10% SDS/PAGE and transferred to nitrocellulose membrane. We used a human TGF‐β1 antibody (RD System, Cat No. AB‐246‐NA) and the Smad2/3 Antibody Sampler Kit (Cell Signaling Technology, Beverly, MA, USA, Cat No. 12747). β‐Tubulin was used as a loading control (Cell Signaling Technology, Cat No. 2146). Densitometric analyses were performed using image j software (NIH, Bethesda, MD, USA).

### Statistical analyses

Statistical analyses were performed using jmp software (SAS Institute Inc, Cary, NC, USA). Expression levels of transcripts were expressed as ΔCT relative to G6PD expression (mean value ± SEM). Comparison of gene expression between the SVF fraction, whole adipose tissue, adipocytes, and DFAT cells was made using ANOVA followed by a Tukey post hoc test. Differences in mRNA expression and protein expression between control and treated cells were tested using matched paired t‐test analyses.

## Results

### Gene expression

**Figure 2:**
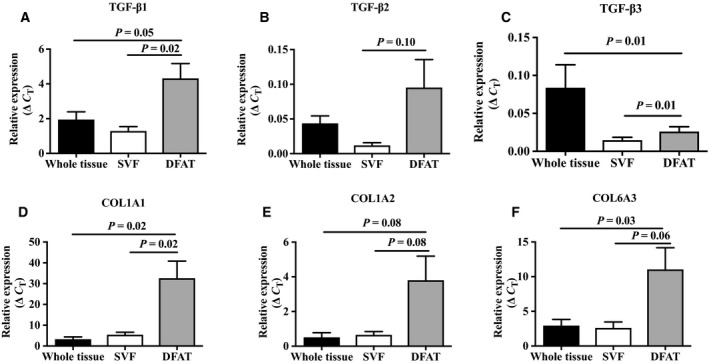
Expression levels of (A) TGF‐β1, (B) TGF‐β2, (C) TGF‐β3, (D) COL1A1, (E) COL1A2, and (F) COL6A3 in whole adipose tissue, SVF, and DFAT cells (day 12) (*n* = 5 donors). Values are mean ± SEM.

We first measured gene expression of TGF‐β1, TGF‐β2, TGF‐β3, COL1A1, COL1A2, and COL6A3 in whole adipose tissue, SVF cells, and DFAT cells by real‐time quantitative RT‐PCR. As shown in Fig. 2, expression of TGF‐β1, TGF‐β3, and COL1A1 was significantly higher in DFAT cells compared to the SVF (*P* = 0.02, *P* = 0.01, and *P* = 0.02, respectively), whereas trends were observed for a similar pattern with TGF‐β2, COL1A2, and COL6A3 (*P* = 0.10, *P* = 0.08, *P* = 0.06, respectively). TGF‐β1, COL1A1, and COL6A3 gene expression was significantly higher in DFAT cells compared to whole adipose tissue (*P* = 0.05, *P* = 0.01, *P* = 0.02, and *P* = 0.03, respectively), and there was a trend for higher expression of COL1A2 in DFAT cells (*P* = 0.08).

**Figure 3:**
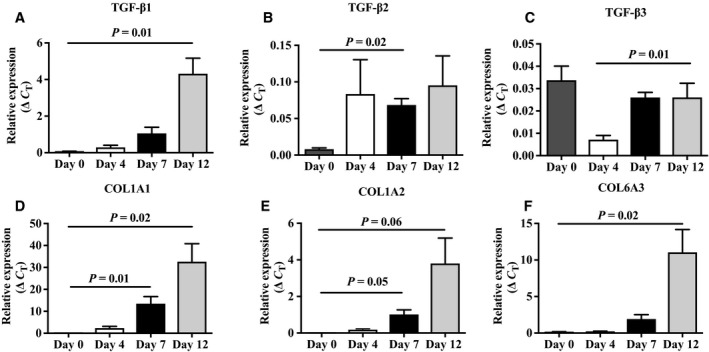
Expression levels of (A) TGF‐β1, (B) TGF‐β2, (C) TGF‐β3, (D) COL1A1, (E) COL1A2, and (F) COL6A3 at various time points during the dedifferentiation process (*n* = 3 donors). Values are mean ± SEM.

We then examined the expression of these transcripts during the dedifferentiation process at day 0, corresponding to mature adipocytes, and at days 4, 7, and 12. As shown in Fig. 3, TGF‐β1, COL1A1, and COL6A3 gene expression increased significantly from day 0 to day 12 of the process (*P* = 0.01, *P* = 0.02, *P* = 0.02). The increase in COL1A2, TGF‐β2 gene expression from day 0 to day 7 was significant (*P* = 0.05, *P* = 0.02, and *P* = 0.02). A trend was observed for higher expression at day 12 compared to day 0 for COL1A2 (*P* = 0.06). Protein expression of TGF‐β1 was confirmed by western blotting at days 0, 4, 7, and 12 in the SC and OM depots (data not shown).

### TGF‐β1 treatment

**Figure 4:**
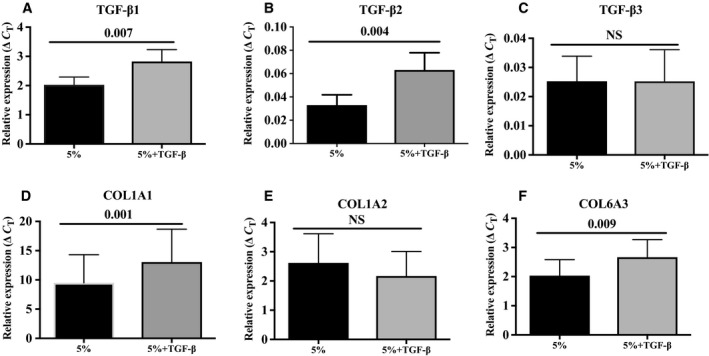
Expression levels of (A) TGF‐β1, (B) TGF‐β2, (C) TGF‐β3, (D) COL1A1, (E) COL1A2, and (F) COL6A3 in dedifferentiating adipocytes incubated in media containing 5% serum or 5% serum supplemented with TGF‐β1 (5 ng·mL^−1^) (*n* = 7 samples). Values are mean ± SEM.

Using the six‐well plate culture system, we tested the effect of serum starvation on the cells. However, when adipocytes were plated in the well without serum, they did not adhere to the upper slides (data not shown). We then used DMEM/F12 supplemented with 5% serum to test the effect of 5 ng·mL^−1^ recombinant TGF‐β1 or vehicle for 24 h on collagen gene expression in DFAT cells from seven patients. As shown in Fig. 4, supplementation with TGF‐β significantly increased TGF‐β1, TGF‐β2, COL1A1, and COL6A3 gene expression compared to 5% serum alone (*P* < 0.05 for all). The treatment had no significant effect on TGF‐β3 and COL1A2 gene expression.

### TGF‐β receptor 1 inhibitor

**Figure 5:**
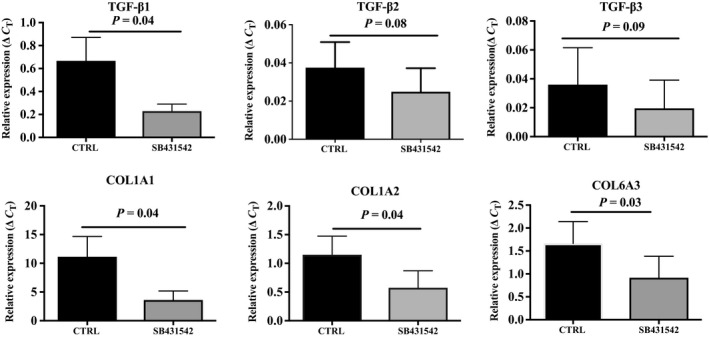
Expression of (A) TGF‐β1, (B) TGF‐β2, (C) TGF‐β3, (D) COL1A1, (E) COL1A2, (F) COL6A3 after treatment with SB431542 or vehicle (CTRL) at day 5 of the dedifferentiation process (*n* = 8 samples). Values are mean ± SEM.

We used our six‐well plate model to investigate whether endogenous inhibition of TGF‐β signaling by SB431542, a TGF‐β receptor ALK5 inhibitor, would downregulate collagen transcripts during the dedifferentiation process. At day 5 of the ceiling culture, cells from *n* = 8 donors were treated with 1 μm SB431542 or with vehicle for 24 h and gene expression was measured by real‐time quantitative RT‐PCR. Fig. 5 shows that treating cells with the inhibitor significantly decreased expression of TGF‐β1, COL1A1, COL1A2, and COL6A3 (*P* = 0.04, *P* = 0.04, *P* = 0.04, and *P* = 0.03, respectively). Trends were observed for a decrease in TGF‐β2 and TGF‐β3 gene expression.

**Figure 6:**
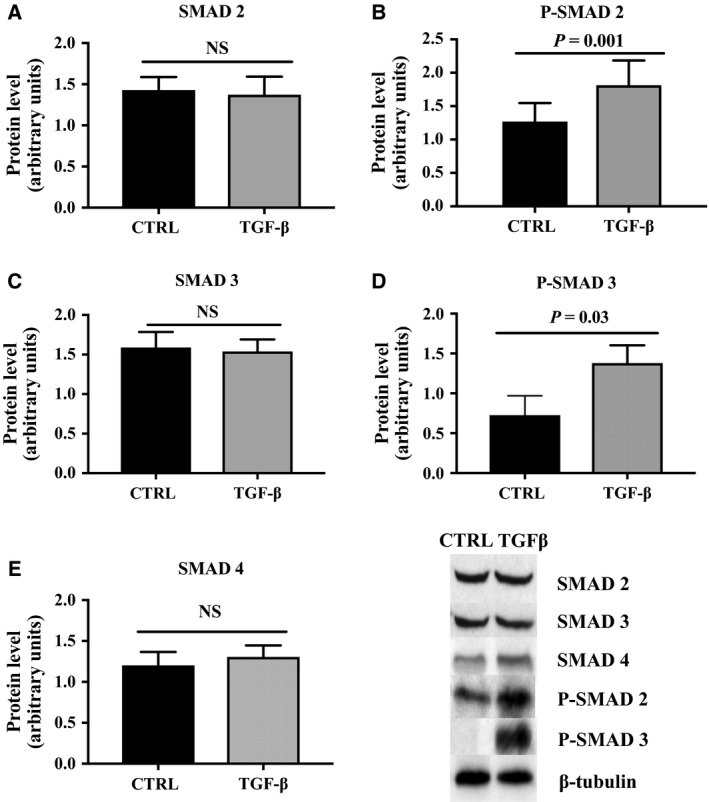
Protein level of (A) SMAD 2, (B) phospho‐SMAD 2, (C) SMAD 3, (D) phospho‐SMAD 3 and (E) SMAD 4 measured in DFAT cells incubated with or without 5 ng·mL^−1^ recombinant TGF‐β1 (P‐SMAD 2 and SMAD 2, *n* = 9; P‐SMAD 3, SMAD 3, and SMAD 2, *n* = 7 samples). Values are mean ± SEM. Protein expression relative to β‐tubulin. Bands from a representative blot are shown.

The activation of the serine/threonine kinase pathway by TGF‐β ligands leads to phosphorylation of some members of the intracellular signaling transduction cascade 18. Protein phosphorylation of SMAD 2 and SMAD 3 as well as SMAD 2, SMAD 3, and SMAD 4 levels was measured using western blot analysis in the DFAT cells incubated with or without (vehicle control) 5 ng·mL^−1^ TGF‐β1. As shown in Fig.**** 6, treatment with TGF‐β1 significantly increased phosphorylation level of SMAD 2 and SMAD 3 (*P* = 0.001 and *P* = 0.03, respectively). Protein levels of SMAD 2, SMAD 3, and SMAD 4 were not affected by TGF‐β treatment.

## Discussion

The aim of this study was to test a new ceiling culture system and modulate the process of dedifferentiation using various incubation conditions targeting the TGF‐β pathway. Adipose tissue expresses various types of collagen other than COL1A1, COL1A2, and COL6A3 19, and the presence of the three subunits of type VI collagen is required for the stable synthesis of collagen VI 20. However, following the results of our previous study demonstrating upregulation of COL1A1, COL1A2, and COL6A3 gene expression during dedifferentiation 21, we chose to examine the effects of TGF‐β on these transcripts specifically. We first demonstrated that TGF‐β1, COL1A1, and COL6A3 gene expression was significantly higher in DFAT cells compared to whole adipose tissue. We also observed a general increase in TGF‐β1, COL1A1, COL1A2, and COL6A3 gene expression over time during dedifferentiation. Using the six‐well plate model, we found that incubation of cells with TGF‐β (5 ng·mL^−1^) during dedifferentiation significantly increased TGF‐β1, TGF‐β2, COL1A1, and COL6A3 gene expression compared to 5% serum alone (*P* < 0.05 for all). Furthermore, when treated with the TGF‐β receptor ALK5 inhibitor, we observed a significant decrease in TGF‐β1, COL1A1, COL1A2, and COL6A3 gene expression during the process, showing that our treatment was effective. Finally, recombinant TGF‐β significantly increased the phosphorylation levels of SMAD 2 and SMAD 3 in DFAT cells.

The six‐well plate model of ceiling culture allowed us to treat cells during the dedifferentiation process by targeting and modulating the TGF‐β pathway. The ceiling culture method was first described by Sugihara *et al*. 4 to study the biology of adipocytes. Ceiling culture has since become the standard strategy to dedifferentiate mature adipocytes. This culture system allows the cells to be maintained in culture for long periods of time and to efficiently proliferate. It has proven useful, but the large number of cells required in flask cultures makes it impossible to study the metabolic, molecular, and secretory attributes of the cells under various incubation conditions. Here, we show that our six‐well plate model could be helpful to understand the physiological process of dedifferentiation and to identify the triggering factors. Some authors have put forward the hypothesis that dedifferentiation is caused by limited gas exchange, high serum concentrations, and cell–plastic contact. However, the six‐well plate model that we have developed allows for gas exchanges, which rules out a predominant effect of hypoxia. On the other hand, the high serum conditions may be an important aspect of the culture because it may contain high concentrations of growth factors. Here, we tested lower serum concentrations (5%) and still observed cell adherence and dedifferentiation. However, when the cells were cultured in serum‐free medium, they could not adhere to the surface. The six‐well plate model represents a relevant approach to examine the impact of various culture environments on the cells.

The response of the cells to the treatments with agonists and antagonists of the TGF‐β pathway using the six‐well plate model supports a role for TGF‐β in modulating expression of extracellular matrix components during dedifferentiation. TGF‐β is a well‐known potent inducer of ECM protein‐coding genes such as fibronectin and collagens 22. This multifunctional cytokine has been described as a key factor in matrix remodeling in various physiological and pathological processes 23, 24, 25, 26, 27. However, to the best of our knowledge, we are the first to describe a role for this pathway in human adipocyte dedifferentiation. Due to the limitations in the amount of material, we could not prove a causal impact of TGF‐β/SMAD signaling during dedifferentiation. However, concomitant with the high expression levels of TGF‐β and collagens in DFAT cells, we show that TGF‐β signaling is effective at the end of the process. The increase in SMAD 2/3 phosphorylation following the treatment with recombinant TGF‐β indeed proves that the pathway remains active once the cells are dedifferentiated. We used high concentrations of a soluble and active TGF‐β. Thus, we omitted the essential activation steps of this pathway. Furthermore, active TGF‐β is cleared from the extracellular space if it does not associate rapidly with surface signaling receptors. However, our data demonstrate that the six‐well plate model allows for detailed characterization of the cells regarding a well‐known pathway, and suggest that it could be used to examine other cell programs during dedifferentiation.

Dedifferentiation contributes to the generation of a proliferative cell population that could be useful in cellular therapy or tissue engineering. Modulating the secretory, molecular, or metabolic characteristics of the cells could potentially increase the efficiency of the process. The extent of dedifferentiation is difficult to measure and we do not have a recognized marker for this process yet. We have previously used cell size of the remaining mature cells, but we had reported that qPCR was, to date, a more sensitive method to detect short‐term changes in the process 16. Cell size has little sensitivity for short‐term incubations and effects that may not dramatically alter the process. Accordingly, we observed changes in collagen gene expression with SB431542, but we were unable to detect qualitative or quantitative changes in the visual aspects of the cultures. More studies are needed to address whether the six‐well plate model can be used to modulate the extent of dedifferentiation.

Some limitations need to be acknowledged. As mentioned, we were unable to address whether TGF‐β can modulate the extent of dedifferentiation. Because TGF‐β1 gene expression was very low at the beginning of the process, it would be surprising that it would induce dedifferentiation, even if a previous study has shown that TGF‐β1 is crucial for the dedifferentiation of cancer cells to cancer stem cells in the context of osteosarcoma 5. It was also demonstrated that TGF‐β1 contributes to the loss of the myofibroblast phenotype 28. As mentioned, TGF‐β1 inhibits adipogenesis 8, 10 and we cannot rule out that the treatment with TGF‐β1 increased the proliferative rate of DFAT cells. The observed increase in TGF‐β signaling and collagen gene expression could reflect cell composition of the culture. As the dedifferentiation process takes place, an increasing number of fibroblasts could contribute to the increase in TGF‐β secretion and signaling in DFAT cells. The changes observed in collagen gene expression following the treatment with TGF‐β1 or with TGF‐β signaling inhibitor may indirectly suggest a modulation of fibroblast proliferation.

In conclusion, the six‐well plate culture system will help understand and modulate the dedifferentiation process longitudinally instead of focusing exclusively on the resulting DFAT cells. To the best of our knowledge, this study is also the first to show a role for TGF‐β and the collagens during human mature adipocyte dedifferentiation *in vitro*. Our findings could potentially contribute to a more extensive characterization of the dedifferentiation process with significant interest for tissue engineering and cell‐based therapy.

## Author contributions

JAC was involved in data acquisition, analysis and interpretation of data, manuscript writing, revision of the manuscript, and final approval. JL and MP were involved in data acquisition, analysis and interpretation of data, revision of the manuscript, and final approval. SM and OL were involved in clinical aspects, sample acquisition, review of the manuscript, and approval. JF was involved in interpretation of data, revision of the manuscript, final approval, and study supervision. AT obtained study funding, was involved in design and conduction of the study, data collection and analysis, interpretation of data, manuscript writing, revision of the manuscript, final approval, and study supervision.

## Conflict of interest

AT receives research funding from Johnson & Johnson Medical Companies for studies unrelated to this manuscript.
